# Association between the systemic immune inflammation index and periodontitis: a cross-sectional study

**DOI:** 10.1186/s12967-024-04888-3

**Published:** 2024-01-23

**Authors:** Junfeng Guo, Rufu Xu, Rongxing Liu, Wenjing Lai, Changpeng Hu, Haitao He, Gang Zhang, Guobing Li, Weiwei Zheng, Rong Zhang

**Affiliations:** 1 Department of Pharmacy, Xinqiao Hospital, Third Military Medical University, Chongqing, China; 2 Department of Stomatology, The 970th Hospital of the Joint Logistics Support Force, Yantai, China; 3 Department of Stomatology, Xinqiao Hospital, Third Military Medical University, Chongqing, China

**Keywords:** Periodontitis, Public health, Cross-sectional study, National Health and Nutrition Examination Survey (NHANES)

## Abstract

**Background:**

Periodontitis is a chronic oral inflammatory disease that seriously affects people's quality of life. The purpose of our study was to investigate the correlation between the systemic immune inflammation index (SII) and periodontitis by utilizing a large national survey. This will establish a reference for the early identification and management of periodontitis.

**Methods:**

This study comprised the adult US population who participated in a national periodontitis surveillance project during the six years from 2009 to 2014. Through the utilization of univariate and multivariate weighted logistic regression, we investigated the correlation between the systemic immune inflammation index and periodontitis. Additionally, we employed sensitivity analyses to evaluate the robustness of our findings.

**Results:**

The study involved 10,366 participants with an average age of 51.00 years, of whom 49.45% were male (N = 5126) and 50.55% were female (N = 5240). The prevalence of periodontitis is estimated to be about 38.43% in the US adults aged 30 or older population. Our logistic regression models indicated a positive association between a SII higher than 978 × 10^9^/L and periodontitis. The elder group (aged 50 or older) with SII higher than 978 × 10^9^/L demonstrated a significant correlation with periodontitis in the fully adjusted model (Odds Ratio [OR] = 1.409, 95% Confidence Interval [CI] 1.037, 1.915, *P* = 0.022). However, there is no statistical difference among adults aged 30 to 50. The robustness of our findings was confirmed through sensitivity analyses.

**Conclusions:**

Our study highlights that SII is associated with periodontitis in a nationally representative sample of US adults. And the SII is significantly associated with a high risk of periodontitis in individuals aged 50 or older.

**Supplementary Information:**

The online version contains supplementary material available at 10.1186/s12967-024-04888-3.

## Background

Periodontitis is a prevalent oral disease, affecting up to 50% of the global population [1]. The latest study suggests that periodontitis is caused by an imbalance in the normal bacterial community in the mouth, which disrupts the healthy relationship between the host and microorganisms at the oral barrier, rather than caused by specific pathogens [2]. As a result of dysbiosis within the oral microbial community, the immune response of the body is activated at the oral barrier, leading to a series of inflammatory reactions [3, 4]. The most common symptoms of this disease are inflammation of the soft tissues surrounding the teeth and gradual loss of alveolar bone and periodontal ligaments, which results in edentulism, masticatory dysfunction, and tooth loss [5]. Moreover, a wealth of evidence has shown potential correlations between periodontitis and several chronic non-communicable diseases, such as coronary heart disease, myocardial infarction, stroke, cancer, hypertension, hyperlipidemia, and diabetes [6–12]. Periodontitis may consequently lead to nutritional deficits, reduced self-esteem, and a lower quality of life [13]. With the high rate of occurrence and its potential adverse effects mentioned above, periodontal health is globally considered to be extremely beneficial to public health and financial burden [14].

The systemic immune-inflammation index (SII) has recently been investigated as a new marker for inflammation and prognosis [15]. SII contains information about three different cell types (lymphocyte, neutrophil, and platelet), which has the potential to be a useful tool for studying inflammation [16]. Lymphocytes are associated with bone loss in periodontitis [17]. Neutrophils play an important role in maintaining periodontal tissue homeostasis and defending against acute inflammation [18]. Platelets have the ability to interact with neutrophils and promote the formation of neutrophil extracellular traps to combat pathogenic challenges [19]. Since SII is a comprehensive indicator based on platelet, neutrophil, and peripheral blood lymphocyte counts, it may more accurately represent the equilibrium of the host's inflammatory and immunological conditions [16]. And it has been proven to be an effective marker for predicting unfavorable clinical outcomes in patients suffering from cancer and inflammatory diseases [20]. Furthermore, recent studies revealed a correlation between SII and generalized stage III grade C periodontitis in young adults [21] and a J-shaped relationship between SII and periodontitis in US adults [22]. However, there is no evidence to show that SII is correlated to the risk of periodontitis in the older population.

In this study, multivariate weighted logistic regression and sensitivity analyses were employed to uncovered the correlation between SII and the risk of periodontitis. The source data were downloaded from the National Health and Nutrition Examination Survey (NHANES) database. NHANES have implemented a full-mouth periodontal examination (FMPE) protocol from 2009 to 2014. This protocol involves taking probing measures from six different sites around each tooth, excluding third molars, and was conducted to evaluate the periodontal health of adults over the age of 30 in the United States. Our study revealed that SII was significantly associated with a high risk of periodontitis among US adults aged 50 or older, and it suggested that SII might be an ideal marker for periodontitis.

## Methods

### Data sources and study population

**Fig. 1:**
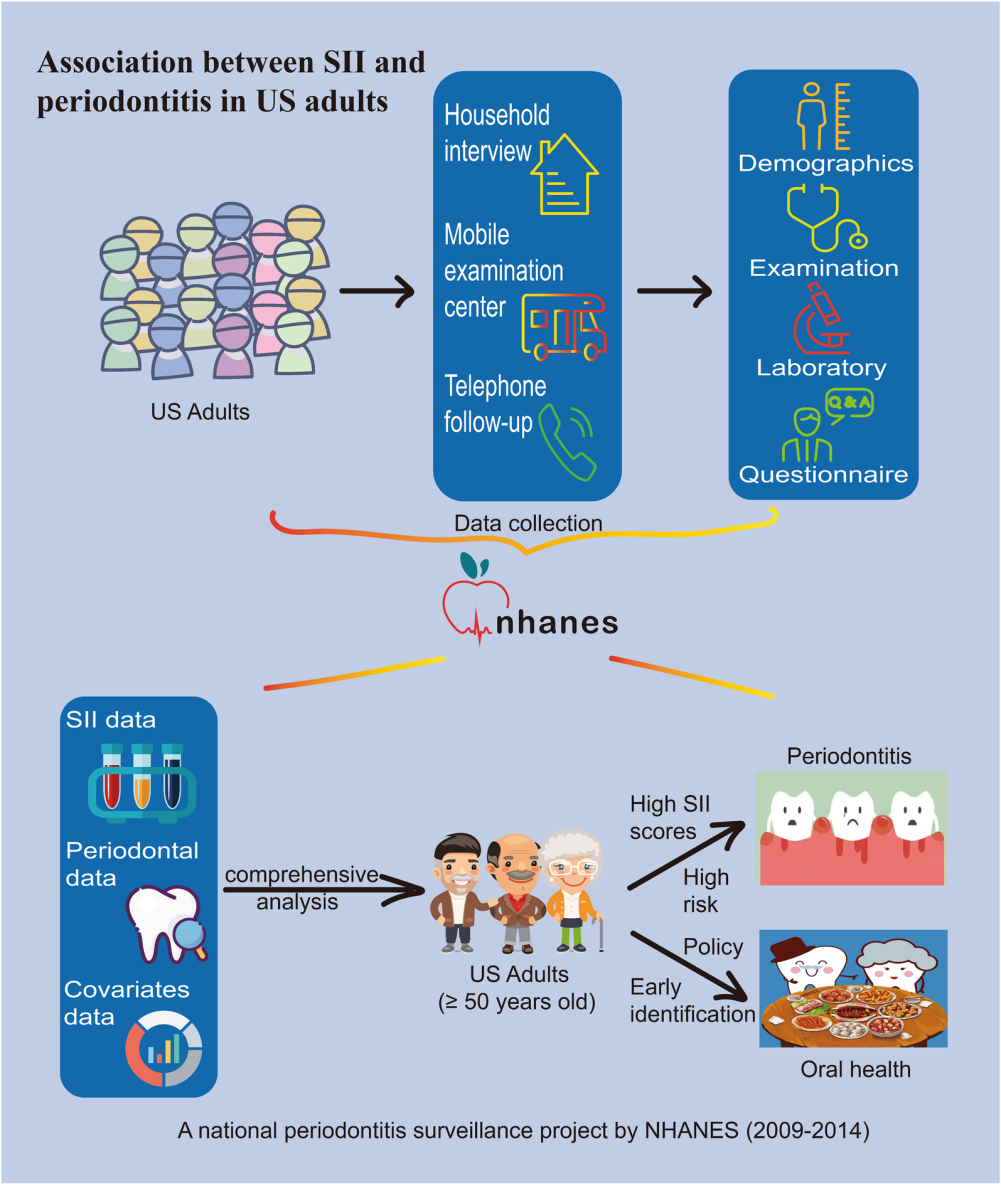
Overview of our study

Our data comes from the NHANES database, which contains studies to examine the health and nutritional well-being of adults and children in the United States. The survey is conducted annually on a representative sample of around 5,000 people in US. These folks are distributed throughout the country, of which 15 counties are visited annually. During the NHANES interview, the demographic information, socioeconomic status, dietary habits, and health-related concerns of participants were collected. NHANES launched a six-year national periodontitis surveillance initiative in 2009. This project involved a FMPE study on individuals aged 30 or older, including assessment of the extent of gingival recession and measurement of their pocket depths. The National Center for Health Statistics' Ethics Review Board approved the study, which was conducted with the explicit written consent of all participants. Overview of our study design in Fig. 1.

Our study enrolled a total of 10,366 participants, spanning from 2009 to 2014. The following participants were excluded from our study: (1) incomplete data of platelets, neutrophils, and lymphocytes (N = 5219). (2) incomplete data of periodontitis (N = 13851). (3) incomplete data of covariates (N = 1032). Finally, we were able to enroll a sizeable sample of American adults with periodontitis (N = 10366), which was analyzed as a representative population of the entire nation.

### Ascertainment of periodontitis

The classical method of diagnosing periodontitis involves assessing clinical parameters such as the plaque index, periodontal pocket probing, bleeding on probing, and the clinical attachment levels [23]. Mild periodontitis was defined as having ≥ 2 interproximal sites with attachment loss (AL) ≥ 3 mm and ≥ 2 interproximal sites with probing depth (PD) ≥ 4 mm (not on the same tooth), or one site with PD ≥ 5 mm. Moderate periodontitis was defined as having ≥ 2 interproximal sites with AL ≥ 4 mm (not on the same tooth), or ≥ 2 interproximal sites with PD ≥ 5 mm (not on the same tooth). Severe periodontitis was defined as having ≥ 2 interproximal sites with AL ≥ 6 mm (not on the same tooth) and ≥ 1 interproximal site with PD ≥ 5 mm (Table 1). Additionally, for mild, moderate, and severe periodontitis, we describe extent and distribution as localized (< 30% of teeth involved) and generalized (≥ 30% of teeth involved) periodontitis [24].

**Table 1:** Diagnostic criteria for different degrees of periodontitis

Periodontitis	Diagnostic criteria
No	No evidence of mild, moderate, or severe periodontitis
Mild	≥ 2 interproximal sites with AL ≥ 3 mm and ≥ 2 interproximal sites with PD ≥ 4 mm (not on the same tooth), or one site with PD ≥ 5 mm
Moderate	≥ 2 interproximal sites with AL ≥ 4 mm (not on the same tooth), or ≥ 2 interproximal sites with PD ≥ 5 mm (not on the same tooth)
Severe	≥ 2 interproximal sites with AL ≥ 6 mm (not on the same tooth) and ≥ 1 interproximal site with PD ≥ 5 mm

AL: attachment loss, PD: probing depth

### Definition of systemic immune inflammation index

Individual cell counts (neutrophils, lymphocytes, and platelets) from the NHANES were used to calculate the SII for the study population. The SII was calculated using a formula described in previous research papers (SII = platelet count × neutrophil count / lymphocyte count) [16]. The methods used to derive complete blood count parameters are based on the Beckman Coulter method of counting and sizing, and combination with an automatic diluting and mixing device for sample processing, and a single-beam photometer for hemoglobinometry. For more information, refer to Chapter 7 of the NHANES Laboratory/Medical Technologists Procedures Manual. We determined the optimal cutoff value of the SII level by using the receiver operating characteristics (ROC) curve.

### Assessment of covariates

We have selected the following variables as probable covariates in our study, as there are some extraneous factors that may impact the outcomes. Demographic data include gender (male or female), age (30–50 years or ≥ 50 years), race (Non-Hispanic White, Non-Hispanic Black, Mexican American, other Hispanic, or other races), education level (less than high school, high school, or above high school), and income to poverty ratio (PIR < 1.3 or PIR ≥ 1.3). The questionnaire data include alcohol consumption (1–10 drinks/month, 10–20 drinks/month, 20 + drinks/month, or non-drinker), smoking status (former smoker, current smoker, or never smoker), coronary heart disease (yes or no), myocardial infarction (yes or no), stroke (yes or no), cancer (yes or no), hypertension (yes or no), hypercholesterolemia (yes or no), and diabetes (yes or no). Examination data include the body mass index (BMI < 25, 25 to < 30, ≥ 30).

### Statistical analyses

To compare the SII subgroups, a survey-weighted logistic regression was used for continuous variables (mean ± SD) and a survey-weighted Chi-square test was used for categorical variables (n count). The correlation between SII and periodontitis was investigated through univariate and multivariate logistic regression analyses. In order to incorporate covariate adjustments, three models were developed. In Model 1, no adjustments were made for any covariates. In Model 2, adjustments were made for gender, age, race, education level, PIR, BMI, alcohol consumption, and smoking status. In Model 3, we made adjustments for systemic diseases that had previously been reported to be related to periodontitis, including coronary heart disease, myocardial infarction, stroke, cancer, hypertension, hypercholesterolemia, and diabetes [6–12]. A comprehensive stratified logistic regression analysis was performed to identify the variables that influence the correlation among participants aged 50 or older. A sensitivity analysis was further conducted using multiple imputation approaches. Missing data was imputed through the use of a multiple imputation chain-equation [25].

All statistical analyses were conducted using R (version 4.2.2). A p-value of less than 0.05 was considered significant for two-tailed tests.

## Results

### Study population characteristics of NHANES

**Fig. 2:**
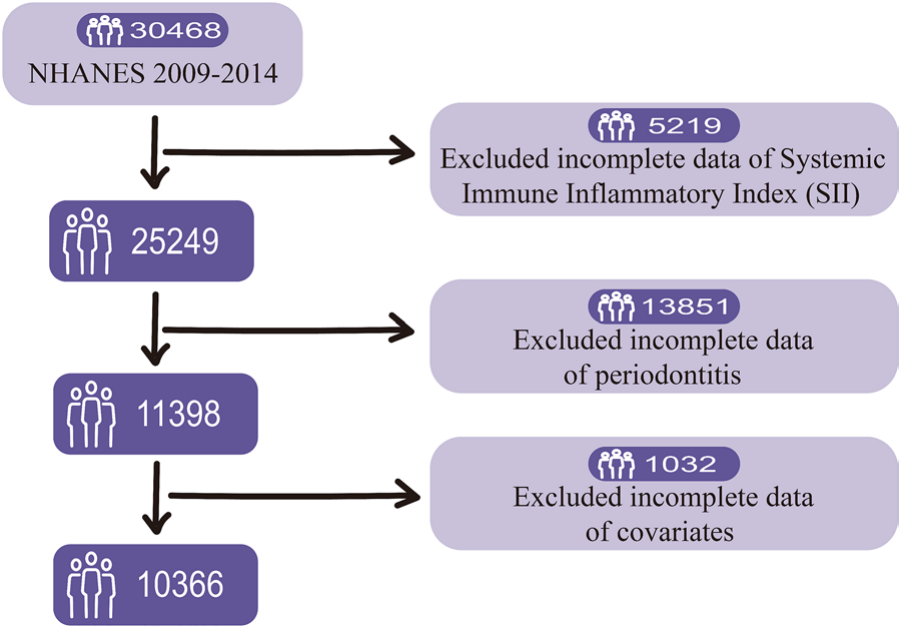
Flowchart of the participants selection from NHANES 2009–2014

A total of 30,468 individuals were involved in the national periodontitis surveillance project conducted by NHANES from 2009 to 2014. According to NHANES rules, participants who were 30 years or older and had at least one tooth (excluding third molars) and did not fulfill any of the health exclusion criteria were eligible for the periodontal evaluation. Our study eventually included 10,366 participants after excluding 5129 participants who lacked complete data on their platelet count, neutrophil count, and lymphocyte count, along with 13,851 participants who lacked complete data on periodontitis and 1032 participants who lacked complete data on covariates (Fig. 2). The study population consisted of 5126 males (49.45% of the total) and 5240 females (50.55%). The average age of the study population was 51.00 years. The weighted study population represents adults aged 30 or older throughout the United States. Periodontitis was diagnosed in 3,984 participants, accounting for 38.43% of the total number of participants. Among them, there were 428 participants with mild periodontitis (including 268 with localized and 160 with generalized periodontitis), 2861 participants with moderate periodontitis (including 1,554 with localized and 1307 with generalized periodontitis), and 695 participants with severe periodontitis (all with generalized periodontitis).We found that there was no statistical difference in SII variation between mild, moderate, and severe periodontitis (*P* > 0.05, Additional file 1: Table S1), or between localized and generalized periodontitis (*P* > 0.05, Additional file 2: Table S2). The median of SII in mild, moderate, and severe periodontitis was significantly higher than that of participants without periodontitis (*P* < 0.01, Additional file 1: Table S1). Therefore, the periodontal status is divided into two groups: those with mild, moderate, or severe periodontitis (classified as "yes") and those without (classified as "no"). The optimal cutoff for SII level was 978 × 10^9^/L (AUC = 0.635, 95% CI: 0.624—0.645, *P* < 0.01, Additional file 6: Figure S1). Furthermore, it has been noted that individuals with high SII levels often have neutrophilia, lymphopenia, or thrombocytosis [26]. We found that participants with high SII had higher neutrophil counts, lower lymphocyte counts, and higher platelet counts (*P* < 0.01, Additional file 3: Table S3).

The clinical characteristics of the participants were analyzed according to their periodontal status, as presented in Table 2. The results demonstrate a statistically significant disparity in gender, age, race, education level, PIR, alcohol consumption, smoking status, high SII scores, coronary heart disease, myocardial infarction, hypertension, and diabetes (all *P* < 0.05).

**Table 2:** Baseline characteristics of participants with different periodontal status

Characteristics	Overall^1^ N = 10,366	Periodontitis^1^		*P* value
		Yes N = 3,984	No N = 6,382	
Gender				< 0.001
Male	5,126 (49.45%)	2,378 (59.69%)	2,748 (43.06%)	
Female	5,240 (50.55%)	1,606 (40.31%)	3,634 (56.94%)	
Age				< 0.001
30–50 years	4,791 (46.22%)	1,448 (36.35%)	3,343 (52.38%)	
≥ 50 years	5,575 (53.78%)	2,536 (63.65%)	3,039 (47.62%)	
Race				< 0.001
Non-Hispanic White	4,774 (46.05%)	1,484 (37.25%)	3,290 (51.55%)	
Non-Hispanic Black	2,108 (20.34%)	989 (24.82%)	1,119 (17.53%)	
Mexican American	1,345 (12.98%)	692 (17.37%)	653 (10.23%)	
Other Hispanic	958 (9.24%)	378 (9.49%)	580 (9.09%)	
Other Races	1,181 (11.39%)	441 (11.07%)	740 (11.60%)	
Education level				< 0.001
Less than High school	2,508 (24.22%)	1,271 (31.95%)	1,237 (19.40%)	
High school	2,291 (22.12%)	1,007 (25.31%)	1,284 (20.13%)	
Above high school	5,556 (53.66%)	1,700 (42.74%)	3,856 (60.47%)	
PIR				< 0.001
Low (< 1.3)	3,275 (31.59%)	1,556 (39.06%)	1,719 (26.94%)	
Mid-high (≥ 1.3)	7,091 (68.41%)	2,428 (60.94%)	4,663 (73.06%)	
Alcohol consumption				< 0.001
1–10 drinks/month	5,538 (57.16%)	2,047 (54.94%)	3,491 (58.54%)	
10–20 drinks/month	700 (7.22%)	272 (7.30%)	428 (7.18%)	
20 + drinks/month	849 (8.76%)	403 (10.82%)	446 (7.48%)	
Non-drinker	2,602 (26.86%)	1,004 (26.95%)	1,598 (26.80%)	
Smoking status				< 0.001
Former smoker	2,753 (26.57%)	1,112 (27.93%)	1,641 (25.72%)	
Current smoker	2,064 (19.92%)	1,064 (26.73%)	1,000 (15.67%)	
Never smoker	5,545 (53.51%)	1,805 (45.34%)	3,740 (58.61%)	
BMI				0.999
Normal(< 25)	2,772 (26.74%)	1,066 (26.76%)	1,706 (26.73%)	
Overweight(25 to < 30)	3,557 (34.31%)	1,366 (34.29%)	2,191 (34.33%)	
Obese(≥ 30)	4,037 (38.94%)	1,552 (38.96%)	2,485 (38.94%)	
SII cutoff				0.002
< 978 × 10^9^/L	9,633 (92.93%)	3669 (92.09%)	5,964(93.45%)	
≥ 978 × 10^9^/L	733 (7.07%)	315 (7.91%)	418 (6.55%)	
Coronary heart disease				0.001
Yes	376 (3.64%)	174 (4.39%)	202 (3.17%)	
No	9,958 (96.36%)	3,791 (95.61%)	6,167 (96.83%)	
Myocardial infarction				0.013
Yes	409 (3.95%)	181 (4.55%)	228 (3.57%)	
No	9,947 (96.05%)	3,797 (95.45%)	6,150 (96.43%)	
Stroke				0.100
Yes	389 (3.75%)	165 (4.14%)	224 (3.51%)	
No	9,971 (96.25%)	3,817 (95.86%)	6,154 (96.49%)	
Cancer				0.868
Yes	1,058 (10.21%)	409 (10.27%)	649 (10.17%)	
No	9,303 (89.79%)	3,572 (89.73%)	5,731 (89.83%)	
Hypertension				< 0.001
Yes	4,123 (39.82%)	1,747 (43.94%)	2,376 (37.26%)	
No	6,230 (60.18%)	2,229 (56.06%)	4,001 (62.74%)	
Hypercholesterolemia				0.114
Yes	3,945 (41.45%)	1,509 (42.48%)	2,436 (40.83%)	
No	5,573 (58.55%)	2,043 (57.52%)	3,530 (59.17%)	
Diabetes				< 0.001
Yes	1,432 (14.22%)	667 (17.28%)	765 (12.32%)	
No	8,637 (85.78%)	3,192 (82.72%)	5,445 (87.68%)	

BMI: body mass index; PIR: Income to poverty ratio; SII: systemic immune inflammatory index

^1^N (unweighted) (%)

### Univariate logistic regression analysis of periodontitis

The results in Table 3 indicated that the risk of suffering from periodontitis is significantly higher among participants who are aged 50 or older, smokers, are non-Hispanic Black, Mexican American, other Hispanic, or other races, have coronary heart disease, have myocardial infarction, have hypertension, have diabetes, and have high SII scores (OR > 1, *P* < 0.05). However, the risk of periodontitis is reduced in participants who are female, have a higher level of education, and have a PIR of 1.3 or above (OR < 1, *P* < 0.05).

**Table 3:** Weighted univariate logistic analysis of periodontitis

Characteristic	OR	95% CI	*P* value
Gender			
Male	Reference	Reference	
Female	0.511	0.471, 0.553	< 0.001
Age			
30–50 years	Reference	Reference	
≥ 50 years	1.927	1.776, 2.089	< 0.001
Race			
Non-Hispanic White	Reference	Reference	
Non-Hispanic Black	2.103	1.698, 2.604	< 0.001
Mexican American	2.419	1.899, 3.081	< 0.001
Other Hispanic	1.702	1.354, 2.140	< 0.001
Other Races	1.641	1.246, 2.161	0.001
Education level			
Less than High school	Reference	Reference	
High school	0.920	0.750, 1.128	0.405
Above high school	0.642	0.538, 0.766	< 0.001
PIR			
Low (< 1.3)	Reference	Reference	
Mid-high (≥ 1.3)	0.575	0.529, 0.626	< 0.001
Alcohol consumption			
Non-drinker	Reference	Reference	
1–10 drinks/month	0.858	0.709, 1.038	0.110
10–20 drinks/month	1.012	0.852, 1.200	0.881
20 + drinks/month	1.163	0.848, 1.596	0.333
Smoking status			
Never smoker	Reference	Reference	
Former smoker	1.372	1.160, 1.622	< 0.001
Current smoker	2.602	2.184, 3.099	< 0.001
BMI			
Normal(< 25)	Reference	Reference	
Overweight(25 to < 30)	0.910	0.736, 1.126	0.369
Obese(≥ 30)	1.051	0.845, 1.307	0.642
SII cutoff			
< 978 × 10^9^/L	Reference	Reference	
≥ 978 × 10^9^/L	1.225	1.052, 1.426	0.009
Coronary heart disease			
No	Reference	Reference	
Yes	1.401	1.140, 1.723	0.001
Myocardial infarction			
No	Reference	Reference	
Yes	1.286	1.054, 1.569	0.013
Stroke			
No	Reference	Reference	
Yes	1.188	0.967, 1.458	0.100
Cancer			
No	Reference	Reference	
Yes	1.011	0.887, 1.152	0.868
Hypertension			
No	Reference	Reference	
Yes	1.320	1.218, 1.431	< 0.001
Hypercholesterolemia			
No	Reference	Reference	
Yes	1.070	0.984, 1.164	0.114
Diabetes			
No	Reference	Reference	
Yes	1.487	1.329, 1.665	< 0.001

BMI: body mass index; CI: confidence interval; OR: odds ratio; PIR: Income to poverty ratio; SII: systemic immune inflammatory index

### Association between SII and periodontitis

The findings display in Table 4 revealed a significant difference between SII and periodontitis in three weighted logistic regression models (OR > 1, *P* < 0.05). Participants with a SII greater than 978 × 10^9^/L are more likely to develop periodontitis. The close correlation between age and periodontitis is widely recognized, and Table 4 also offers additional insight after breaking down the ages of the participants. Logistic regression analysis showed that there was no evidence in Models 1–3 that a SII above 978 × 10^9^/L is associated with a higher risk of periodontitis in the 30–50 years old age group (Model 1: OR = 1.301, 95% CI 0.849, 1.993, *P* = 0.215; Model 2: OR = 1.266, 95% CI 0.768, 2.086, *P* = 0.337; Model 3: OR = 1.213, 95% CI 0.722, 2.039, *P* = 0.445). Finally, a significant association between a high SII and periodontitis was observed in the subgroup of participants who were aged 50 or older after adjusting for all potential confounding factors (Model 3: OR = 1.409, 95% CI 1.037, 1.915, *P* = 0.022).

**Table 4:** Weighted association between SII and periodontitis

Age stratification	SII (10^9^/L)		*P* value
	< 978 (OR, 95% CI)	≥ 978 (OR, 95% CI)	
Overall			
Model 1	Reference	1.444 (1.108, 1.881)	0.005
Model 2	Reference	1.374 (1.067, 1.769)	0.011
Model 3	Reference	1.345 (1.038, 1.743)	0.019
30–50 years old			
Model 1	Reference	1.301 (0.849, 1.993)	0.215
Model 2	Reference	1.266 (0.768, 2.086)	0.337
Model 3	Reference	1.213 (0.722, 2.039)	0.445
≥ 50 years old			
Model 1	Reference	1.485 (1.072, 2.058)	0.015
Model 2	Reference	1.406 (1.037, 1.905)	0.023
Model 3	Reference	1.409 (1.037, 1.915)	0.022

CI: confidence interval; OR: odds ratio; SII: systemic immune inflammatory index

Subsequently, a stratified logistic regression analysis was conducted on participants aged 50 or older, and it was found that there were no factors that could affect the relationship between SII and periodontitis in this age group (Additional file 4: Table S4). In order to ensure the stability of our findings, we employed multiple imputation methods to impute any missing data and conducted a sensitivity analysis. The distribution of characters in the baseline was shown in Additional file 5: Table S5. The sensitivity analysis results revealed that the model, which was adjusted for all potential variables among the participants aged 50 or older, generated consistent results to the aforementioned conclusion (OR = 1.382, 95% CI 1.019, 1.876, *P* = 0.029, Table 5).

**Table 5:** Weighted multivariate logistic analysis and multiple imputation analysis for identifying sensitivity

Age stratification	SII < 978 × 10^9^/L (OR, 95% CI)	SII ≥ 978 × 10^9^/L (OR, 95% CI), *P* value	
		Complete case	Multiple imputation
30–50 years old			
Model 3	Reference	1.213 (0.722, 2.039), 0.445	1.128 (0.669, 1.902), 0.634
≥ 50 years old			
Model 3	Reference	1.409 (1.037, 1.915), 0.022	1.382 (1.019, 1.876), 0.029

CI: confidence interval; OR: odds ratio; SII: systemic immune inflammatory index

## Discussion

Periodontitis is a prevalent and chronic dental disease caused by the disrupted interplay between an imbalanced oral microbiota and the host's immune system [27]. This causes a long-term and progressive degradation of the tissues that support the periodontium (such as the gingiva, cementum, periodontal ligament, and alveolar bone), ultimately resulting in inflammatory bone loss [28]. Periodontitis is the predominant factor for tooth loss among older individuals [29]. Dental practitioners will be attending to care for more older people than in the past due to the growing prevalence of aging populations. Globally, the prevalence of periodontitis has been rising and is still a serious problem [1]. To tackle the worldwide public health issue of periodontitis, it is imperative that policy adjustments be taken at the earliest stage of periodontitis.

Controlling the immune response to suspected periodontal infections has become increasingly important in resolving inflammation, managing the osteolytic environment, and promoting healthy bone growth [30]. Numerous immune cells are activated during periodontitis progresses, adjusting the immune response by generating cytokines and growth factors that affect the activity of bone cells like osteoclasts and osteoblasts [31]. Inflammatory mediators generated from periodontal tissues can simultaneously activate the immune system and initiate a systemic acute-phase response [32]. Important insights into the presence of systemic and periodontal infections can be derived by evaluating systemic circulatory markers such as lymphocyte, neutrophil, platelet, and erythrocyte counts [33–37]. Several studies have shown that the neutrophil–lymphocyte ratio (NLR), the platelet-lymphocyte ratio (PLR), and the lymphocyte-monocyte ratio (LMR) may be potential biomarkers for identifying periodontitis in healthy individuals [21, 38, 39]. We attempted to determine the optimal cutoff values of NLR, PLR, and LMR using ROC curves among the 10,366 participants in our study. However, NLR, PLR, and LMR did not show better predictive validity when compared with SII among participants aged 50 or older (AUC for SII: 0.635, P < 0.01, Additional file 6: Fig. S1; AUC for NLR: 0.493, P > 0.05; AUC for PLR: 0.534, P < 0.01; AUC for LMR: 0.512, P < 0.05, Additional file 7: Fig. S2). A meta-analysis showed that serum C-Reactive protein (CRP) levels were closely related to periodontitis [40]. Additionally, it was discovered that the prevalence of periodontitis in the American population increases with increasing CRP levels, but this association only exists in individuals with a BMI greater than 30 kg/m^2^ [41]. We were unable to compare the performance of SII with CRP in predicting periodontitis in adults aged 50 or older due to the missing CRP data for 2011–2014 from the national periodontitis surveillance project NHANES 2009–2014.

SII, a novel index that considers lymphocyte, neutrophil, and platelet counts [16], is commonly utilized for the evaluation of diverse diseases. In bladder cancer patients, SII could serve as a reliable autonomous prognostic predictor for individuals who have had surgery [42]. Individuals exhibiting high SII levels often present with neutrophilia, lymphopenia, or thrombocytosis [22, 26]. This is consistent with the findings of our study. Recent studies have also demonstrated that a high SII is a detrimental prognostic factor for people who have been diagnosed with gastroesophageal adenocarcinoma [43]. Furthermore, SII is frequently used to estimate the presence of systemic diseases such as diabetes [44], ankylosing spondylitis [45], and coronary heart disease [46]. Consequently, we made an effort to employ SII as an assessment measurement for periodontitis. In our study, the large national survey dataset was utilized to investigate the connection between SII and periodontitis. As elderly individuals may have a higher prevalence of systemic diseases [6–12], we accounted for relevant covariates based on existing research and eliminated the influence of such diseases. Our study found that a significant correlation between high SII scores and a high risk of periodontitis in elderly participants. Recently, an Indian researcher conducted a multicenter, hospital-based case–control study from January 2017 to December 2021. They found that young adults with generalized stage III grade C periodontitis are linked to SII [21]. However, our study suggests that participants with high SII scores who are aged 50 or older are more likely to develop periodontitis. In addition, our results were confirmed through sensitivity analyses using multiple imputations. It is possible that the limited number of participants (ultimately included 315 participants) in her investigation contributed to this outcome, whereas our expansive nationwide survey database may have mitigated certain sources of bias, thus serving as an update and supplement to previous studies.

The findings of Loos et al.'s study propose that exploring the parameters associated with lymphocytes could clarify the factors that make individuals more susceptible to and suffer from periodontitis [47]. Recently, neutrophils have been regarded as key players in the development of periodontitis [18]. Another study suggests that individuals with generalized aggressive periodontitis who exhibit a high degree of inflammation typically have a low number of platelets in their peripheral blood [48]. Therefore, the SII possesses strong predictive power, which is calculated based on the counts of lymphocytes, neutrophils, and platelets in peripheral blood. The results of our cross-sectional study indicate that participants aged 50 or older with higher SII scores are independently associated with periodontitis. While no correlation was found among participants aged 30 to 50. In addition, Sensitivity analyses further confirmed the correlation between SII and periodontitis. Hence, SII can serve as a useful biomarker for identifying periodontitis.

It has been proposed that periodontitis is associated with disparities in oral health in affluent areas while posing serious financial challenges for people living in poverty [49]. In low- and middle-income nations, the lack of access to high-quality oral healthcare and the high cost of treatment can cause catastrophic expenditures [50]. Therefore, there is a pressing need for an affordable and easily obtainable marker to identify and treat periodontitis in its early stages. Our results, obtained from the national periodontitis surveillance project NHANES 2009–2014, revealed that the SII is an excellent indicator for periodontitis among participants aged 50 or older. Furthermore, the counts of lymphocytes, neutrophils, and platelets in peripheral blood can obtained through a simple and affordable complete blood count test. Consequently, incorporating SII into dental assessments can facilitate the early detection of periodontitis in elderly, thereby enabling timely intervention to prevent its progression. This can improve the quality of life and happiness of the elderly population, while also contributing to the WHO's '8020' goal of ensuring that individuals aged 80 or older have a minimum of 20 functional teeth.

In this study, we employed classical diagnostic standards for periodontitis to guarantee the authenticity and applicability of the findings. Nonetheless, this study included several inherent flaws and limitations. First, the cross-sectional study design prevented us from determining a causal relationship. Second, because only those aged 30 or older are included in the national periodontal surveillance project, so we lack insight into the periodontal condition of those under 30 years old. Third, despite adjusting for a variety of relevant factors, we were unable to completely exclude the impact of other potential confounding variables. Fourth, SII could only predict the risk of periodontitis in people aged 50 or older, but it could not distinguish the stages of periodontitis. Fifth, due to the lack of oral imaging data of participants in the national periodontitis surveillance project NHANES 2009–2014, the new classification system of periodontitis cannot be used in this study. To corroborate our conclusions, additional prospective investigations on a larger scale are required.

## Conclusions

In summary, SII may serve as a convenient and affordable marker of inflammation that could be employed to anticipate the risk of periodontitis in individuals who are 50 years old or older. With this discovery, it is beneficial in designing and implementing effective dental examination strategies in order to improve the public's oral health.

### Supplementary Information

**Additional file 1: Table S1.** Weighted association between SII and no, mild, moderate, or severe periodontitis.

**Additional file 2: Table S2.** Weighted association between SII and localized or generalized periodontitis.

**Additional file 3: Table S3.** Weighted association between SII and neutrophil count, lymphocyte count, or platelet count.

**Additional file 4: Table S4.** weighted stratified logistic regression analysis of participants aged 50 or older.

**Additional file 5: Table S5.** The basic characteristics of complete cases and cases following multiple imputation.

**Additional file 6: Figure S1.** ROC curves of SII.

**Additional file 7: Figure S2.** ROC curves. **A** ROC curves of NLR, **B** ROC curves of PLR, **C** ROC curves of LMR.

## Data Availability

Publicly available datasets were analyzed in this study. These data can be downloaded from: https://www.cdc.gov/nchs/nhanes/.

## Abbreviations

AL: Attachment loss
BMI: Body mass index
CI: Confidence interval
FMPE: Full-mouth periodontal examination
NHANES: National Health and Nutrition Examination Survey
OR: Odds ratio
PD: Probing depth
PIR: Income to poverty ratio
SII: Systemic immune inflammation index
