# Supplementary material for: Association between the systemic immune inflammation index and periodontitis: a cross-sectional study
Source: J Transl Med. 2024 Jan 23;22:96. doi: 10.1186/s12967-024-04888-3 (PMC10804475; doi:10.1186/s12967-024-04888-3)
Supplement: Supplementary file 1 — Additional file 1: Table S1. Weighted association between SII and no, mild, moderate, or severe periodontitis. [file 12967_2024_4888_MOESM1_ESM.docx]

Table S1. Weighted association between SII and no, mild, moderate, or severe periodontitis

| Periodontitis | SII (10^9^/L), M (Q1, Q3) | *P*_1_ value | *P*_2_ value | *P*_3_ value |
| --- | --- | --- | --- | --- |
| No | 1021 (892, 1200) |  |  |  |
| Mild | 1129 (998, 1316) | <0.001 |  |  |
| Moderate | 1123 (998, 1312) | <0.001 | 0.893 |  |
| Severe | 1125 (990, 1326) | <0.001 | 0.863 | 0.932 |

*P*_1_ value: No vs. Mild, Moderate, or Severe, *P*_2_ value: Mild vs. Moderate, or Severe, *P*_3_ value: Moderate vs. Severe
