# Supplementary material for: Association between the systemic immune inflammation index and periodontitis: a cross-sectional study
Source: J Transl Med. 2024 Jan 23;22:96. doi: 10.1186/s12967-024-04888-3 (PMC10804475; doi:10.1186/s12967-024-04888-3)
Supplement: Supplementary file 2 — Additional file 2: Table S2. Weighted association between SII and localized or generalized periodontitis. [file 12967_2024_4888_MOESM2_ESM.docx]

Table S2. Weighted association between SII and localized or generalized periodontitis

| Periodontitis | SII (10^9^/L) of extent and distribution, M (Q1, Q3) | | *P* value |
| --- | --- | --- | --- |
|  | **Localized** | **Generalized** |  |
| Mild | 1139 (1013, 1315) | 1123 (989, 1319) | 0.336 |
| Moderate | 1119 (998, 1302) | 1131 (1001, 1331) | 0.146 |
| Severe^*^ | - | 1125 (990, 1326) | - |

^*^All participants with severe periodontitis were generalized.
