# Supplementary material for: Association between the systemic immune inflammation index and periodontitis: a cross-sectional study
Source: J Transl Med. 2024 Jan 23;22:96. doi: 10.1186/s12967-024-04888-3 (PMC10804475; doi:10.1186/s12967-024-04888-3)
Supplement: Supplementary file 3 — Additional file 3: Table S3. Weighted association between SII and neutrophil count, lymphocyte count, or platelet count. [file 12967_2024_4888_MOESM3_ESM.docx]

Table S3. Weighted association between SII and neutrophil count, lymphocyte count, or platelet count

| Peripheral blood cells | Cell counts (10^9^/L) of SII subgroup, M (Q1, Q3) | | *P* value |
| --- | --- | --- | --- |
|  | **SII < 978 × 10^9^/L** | **SII ≥ 978 × 10^9^/L** |  |
| Neutrophils | 3.8 (3.0, 4.8) | 6.6 (5.4, 8.1) | <0.001 |
| Lymphocytes | 2.0 (1.6, 2.5) | 1.6 (1.2, 1.9) | <0.001 |
| Platelets | 227 (193, 265) | 290 (246, 344) | <0.001 |
