# Supplementary material for: Association between the systemic immune inflammation index and periodontitis: a cross-sectional study
Source: J Transl Med. 2024 Jan 23;22:96. doi: 10.1186/s12967-024-04888-3 (PMC10804475; doi:10.1186/s12967-024-04888-3)
Supplement: Supplementary file 4 — Additional file 4: Table S4. weighted stratified logistic regression analysis of participants aged 50 or older. [file 12967_2024_4888_MOESM4_ESM.docx]

Table S4. weighted stratified logistic regression analysis of participants aged 50 or older

| Characteristics | SII (10^9^/L) | | *P* value |
| --- | --- | --- | --- |
|  | ＜978 (OR, 95% CI) | ≥ 978 (OR, 95% CI) |  |
| Gender |  |  |  |
| Male | Reference | 1.380 (0.911, 2.092) | 0.111 |
| Female | Reference | 1.326 (0.880, 1.996) | 0.157 |
| Race |  |  |  |
| Non-Hispanic White | Reference | 1.521 (1.058, 2.186) | 0.058 |
| Non-Hispanic Black | Reference | 1.605 (0.854, 3.017) | 0.122 |
| Mexican American | Reference | 1.643 (0.618, 4.370) | 0.192 |
| Other Hispanic | Reference | 0.377 (0.119, 1.200) | 0.073 |
| Other Races | Reference | 0.288 (0.046, 1.796) | 0.145 |
| Education level |  |  |  |
| Less than High school | Reference | 0.804 (0.476, 1.357) | 0.392 |
| High school | Reference | 3.062 (1.514, 6.191) | 0.053 |
| Above high school | Reference | 1.285 (0.895, 1.846) | 0.155 |
| PIR |  |  |  |
| low (< 1.3) | Reference | 1.438 (0.984, 2.103) | 0.069 |
| mid-high (≥ 1.3) | Reference | 1.179 (0.703, 1.979) | 0.513 |
| Alcohol consumption |  |  |  |
| 1-10 drinks/month | Reference | 1.631 (1.095, 2.430) | 0.082 |
| 10-20 drinks/month | Reference | 0.403 (0.083, 1.942) | 0.229 |
| 20+ drinks/month | Reference | 1.995 (1.031, 3.861) | 0.067 |
| Non-drinker | Reference | 1.047 (0.598, 1.834) | 0.867 |
| Smoke status |  |  |  |
| Former smoker | Reference | 1.114 (0.785, 1.581) | 0.526 |
| Current smoker | Reference | 2.156 (0.979, 4.750) | 0.075 |
| Never smoker | Reference | 1.532 (0.857, 2.740) | 0.132 |
| BMI |  |  |  |
| Normal( < 25) | Reference | 2.733 (1.395, 5.353) | 0.062 |
| Overweight(25 to < 30) | Reference | 0.864 (0.549, 1.358) | 0.506 |
| Obese(≥ 30) | Reference | 1.435 (0.893, 2.305) | 0.118 |
| Coronary heart disease |  |  |  |
| Yes | Reference | 1.328 (1.000, 1.762) | 0.060 |
| No | Reference | 2.568 (0.846, 7.797) | 0.077 |
| Myocardial infarction |  |  |  |
| Yes | Reference | 1.304 (0.966, 1.759) | 0.069 |
| No | Reference | 3.847 (1.261, 11.733) | 0.112 |
| Stroke |  |  |  |
| Yes | Reference | 1.187 (0.426, 3.303) | 0.728 |
| No | Reference | 1.398 (1.030, 1.898) | 0.069 |
| Cancer |  |  |  |
| Yes | Reference | 1.320 (0.968, 1.800) | 0.132 |
| No | Reference | 1.600 (0.784, 3.266) | 0.066 |
| Hypertension |  |  |  |
| Yes | Reference | 1.218 (0.767, 1.934) | 0.381 |
| No | Reference | 1.630 (1.007, 2.637) | 0.077 |
| Hypercholesterolemia |  |  |  |
| Yes | Reference | 1.331 (0.835, 2.122) | 0.207 |
| No | Reference | 1.475 (0.945, 2.303) | 0.073 |
| Diabetes |  |  |  |
| Yes | Reference | 1.238 (0.916, 1.674) | 0.145 |
| No | Reference | 2.257 (0.994, 5.126) | 0.083 |

Adjusted for gender, race, education level, PIR, alcohol consumption, smoking status, BMI, coronary heart disease, myocardial infarction, stroke, cancer, hypertension, hypercholesterolemia, and diabetes. All models in each stratification were left unadjusted for the variables they contained.

BMI: body mass index; CI: confidence interval; OR: odds ratio; PIR: Income to poverty ratio; SII: systemic immune inflammatory index.
