# Supplementary material for: Association between the systemic immune inflammation index and periodontitis: a cross-sectional study
Source: J Transl Med. 2024 Jan 23;22:96. doi: 10.1186/s12967-024-04888-3 (PMC10804475; doi:10.1186/s12967-024-04888-3)
Supplement: Supplementary file 5 — Additional file 5: Table S5. The basic characteristics of complete cases and cases following multiple imputation. [file 12967_2024_4888_MOESM5_ESM.docx]

Table S5. The basic characteristics of complete cases and cases following multiple imputation

| Characteristics | Complete case  N^1^=10366 | Multiple imputation  N^1^=11398 |
| --- | --- | --- |
| Gender |  |  |
| male | 5,126 (49.45%) | 5,621 (49.32%) |
| female | 5,240 (50.55%) | 5,777 (50.68%) |
| Age |  |  |
| 30-50 years | 4,791 (46.22%) | 5,224 (45.83%) |
| ≥50 years | 5,575 (53.78%) | 6,174 (54.17%) |
| Race |  |  |
| Non-Hispanic White | 4,774 (46.05%) | 5,073 (44.51%) |
| Non-Hispanic Black | 2,108 (20.34%) | 2,334 (20.48%) |
| Mexican American | 1,345 (12.98%) | 1,546 (13.56%) |
| Other Hispanic | 958 (9.24%) | 1,125 (9.87%) |
| Other Races | 1,181 (11.39%) | 1,320 (11.58%) |
| Education level |  |  |
| Less than High school | 2,508 (24.22%) | 2,879 (25.29%) |
| High school | 2,291 (22.12%) | 2,527 (22.20%) |
| Above high school | 5,556 (53.66%) | 5,978 (52.51%) |
| PIR |  |  |
| low (< 1.3) | 3,275 (31.59%) | 3,312 (31.72%) |
| mid-high (≥ 1.3) | 7,091 (68.41%) | 7,130 (68.28%) |
| Alcohol consumption |  |  |
| 1-10 drinks/month | 5,538 (57.16%) | 6,031 (56.86%) |
| 10-20 drinks/month | 700 (7.22%) | 758 (7.15%) |
| 20+ drinks/month | 849 (8.76%) | 910 (8.58%) |
| Non-drinker | 2,602 (26.86%) | 2,908 (27.42%) |
| Smoke status |  |  |
| Former smoker | 2,753 (26.57%) | 2,995 (26.29%) |
| Current smoker | 2,064 (19.92%) | 2,256 (19.80%) |
| Never smoker | 5,545 (53.51%) | 6,142 (53.91%) |
| BMI |  |  |
| Normal(< 25) | 2,772 (26.74%) | 3,015 (26.66%) |
| Overweight(25 to < 30) | 3,557 (34.31%) | 3,930 (34.75%) |
| Obese(≥ 30) | 4,037 (38.94%) | 4,365 (38.59%) |
| Periodontitis |  |  |
| No | 6,382 (61.57%) | 6,989 (61.32%) |
| mild | 428 (4.13%) | 468 (4.11%) |
| moderate | 2,861 (27.60%) | 3,164 (27.76%) |
| severe | 695 (6.70%) | 777 (6.82%) |
| Coronary heart disease |  |  |
| Yes | 376 (3.64%) | 411 (3.62%) |
| No | 9,958 (96.36%) | 10,949 (96.38%) |
| Myocardial infarction |  |  |
| Yes | 409 (3.95%) | 449 (3.94%) |
| No | 9,947 (96.05%) | 10,935 (96.06%) |
| Stroke |  |  |
| Yes | 389 (3.75%) | 425 (3.73%) |
| No | 9,971 (96.25%) | 10,964 (96.27%) |
| Cancer |  |  |
| Yes | 1,058 (10.21%) | 1,152 (10.12%) |
| No | 9,303 (89.79%) | 10,237 (89.88%) |
| Hypertension |  |  |
| Yes | 4,123 (39.82%) | 4,549 (39.96%) |
| No | 6,230 (60.18%) | 6,834 (60.04%) |
| Hypercholesterolemia |  |  |
| Yes | 3,945 (41.45%) | 4,316 (41.40%) |
| No | 5,573 (58.55%) | 6,109 (58.60%) |
| Diabetes |  |  |
| Yes | 1,432 (14.22%) | 1,591 (14.37%) |
| No | 8,637 (85.78%) | 9,482 (85.63%) |

^1^N (unweighted) (%)

BMI: body mass index; PIR: Income to poverty ratio.
