# Supplementary figures and images for: Association between the systemic immune inflammation index and periodontitis: a cross-sectional study
Source: J Transl Med. 2024 Jan 23;22:96. doi: 10.1186/s12967-024-04888-3 (PMC10804475; doi:10.1186/s12967-024-04888-3)

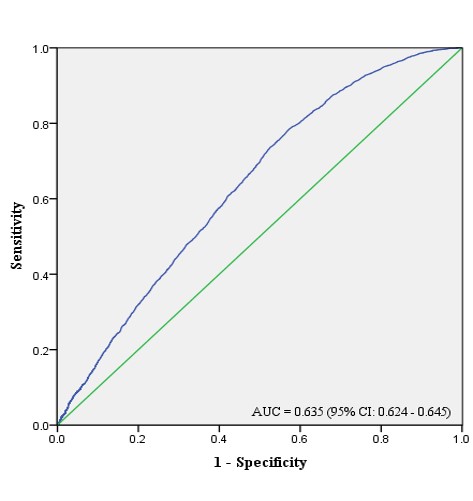

Supplement: Supplementary file 6 — Additional file 6: Figure S1. ROC curves of SII. [file 12967_2024_4888_MOESM6_ESM.jpg]

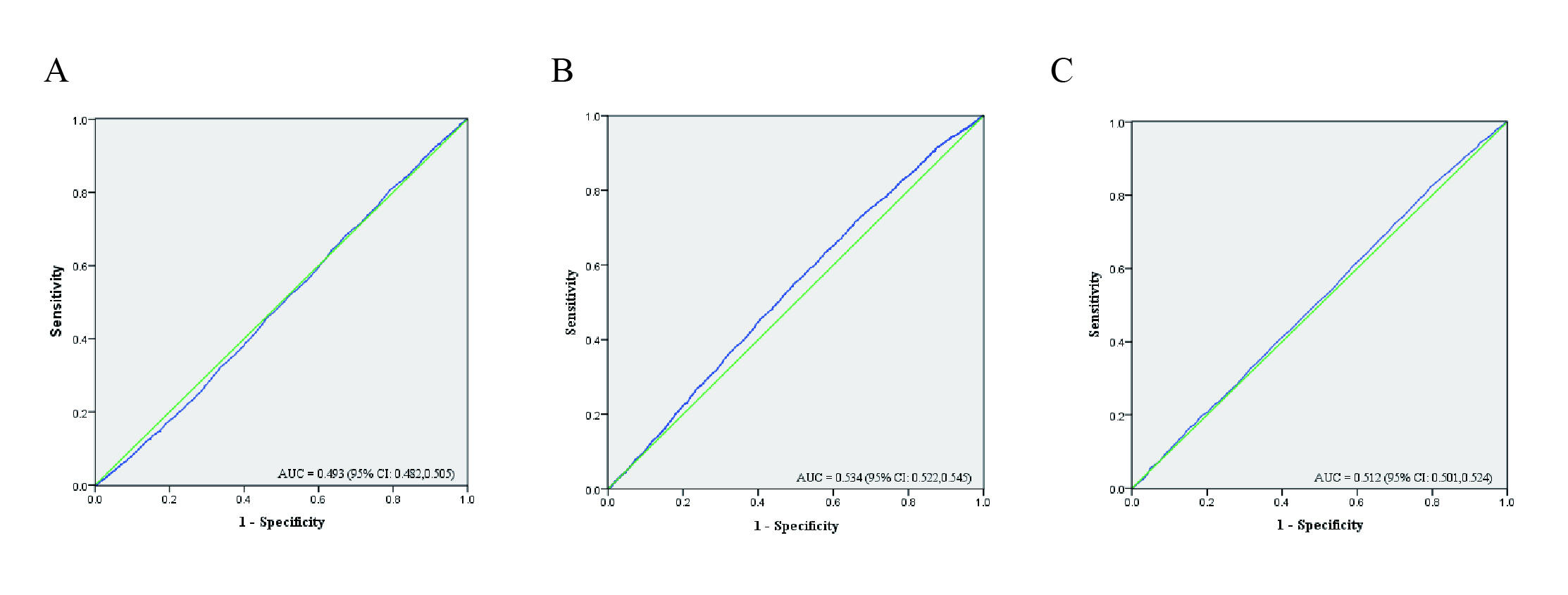

Supplement: Supplementary file 7 — Additional file 7: Figure S2. ROC curves. A ROC curves of NLR, B ROC curves of PLR, C ROC curves of LMR. [file 12967_2024_4888_MOESM7_ESM.jpg]
